# Spontaneous rupture of the extensor pollicis brevis tendon in a baseball pitcher: A case report

**DOI:** 10.1080/03009730902989323

**Published:** 2009-09-07

**Authors:** Takuya Fujimoto, Yoshihiro Tanase, Takashi Oribe, Yasushi Watanabe

**Affiliations:** ^1^Department of Orthopaedic Surgery, Hyogo Cancer CenterAkashiJapan; ^2^Department of Orthopaedic Surgery, Saiseikai Hyogo Prefectural HospitalKobeJapan; ^3^Department of Orthopaedic Surgery, Shinko Kakogawa HospitalKakogawaJapan

**Keywords:** Extensor indicis proprius (EIP), extensor pollicis brevis (EPB), spontaneous rupture, tendon transfer

## Abstract

A 15-year-old pitcher on a boys’ Little League baseball team suffered spontaneous rupture of the extensor pollicis brevis (EPB) tendon. Since the EPB tendon was, at surgery, found hypoplastic and non-functional, transfer of the extensor indicis proprius (EIP) tendon was carried out. After a 6-month period of rehabilitation and follow-up, the patient was able to resume playing baseball. Although rupture of the EPB is rare, transfer of the EIP tendon is one of the treatments of choice for such injuries.

## Introduction

Although the isolated extensor pollicis longus (EPL) rupture is induced by fractures and chronic tendonitis (1,2), rupture of the extensor pollicis brevis (EPB) tendon is very rare. Surgical findings of the isolated spontaneous rupture of the EPB tendon in a right-hand-dominant boy revealed a hypoplastic midsubstance tendon rupture between the metacarpophalangeal (MCP) joint and the first extensor compartment. Here we demonstrate successful treatment of a ruptured EPB tendon with an extensor indicis proprius (EIP) transfer.

## Case report

**Figure 1.**
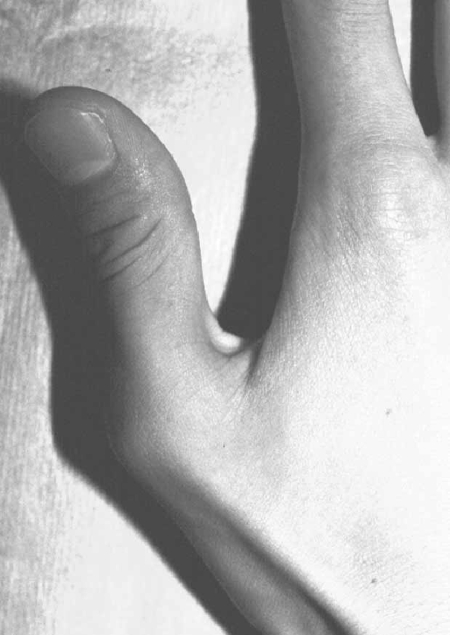
The patient was unable to fully extend the right metacarpophalangeal (MCP) joint.

**Figure 2.**
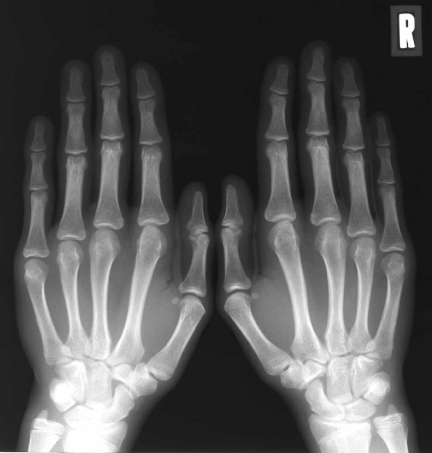
View of the XP photograph of the hand. No abnormalities are evident.

**Figure 3.**
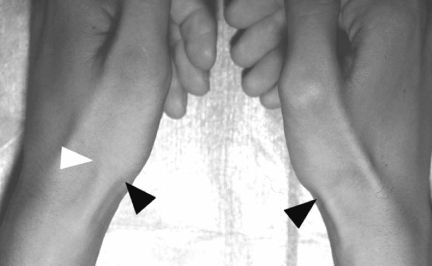
There was no tenderness, swelling, or redness in either hand. The extensor pollicis brevis (EPB) tendon could not be palpated (arrow-head), however. White arrow-head = EPB tendon; black arrow-head = abductor pollicis longus (APL) tendon.

**Figure 4.**
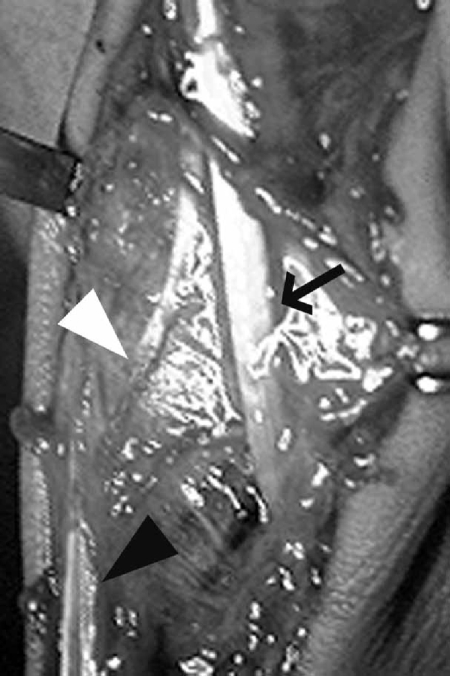
The hypoplastic extensor pollicis brevis (EPB) tendon was found ruptured at the level of the carpometacarpal joint of the right hand. The proximal part of the tendon was absent (arrow-head). The extensor indicis proprius (EIP) was transferred to the base of the proximal phalanx. White arrow-head = EPB tendon; black arrow-head = abductor pollicis longus (APL) tendon; black arrow = EIP tendon.

**Figure 5.**
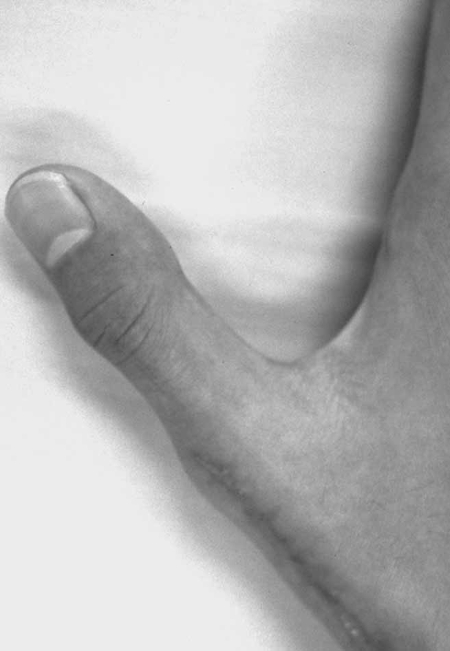
At 6 weeks after the operation, the patient had full range of movement of his right thumb.

A 15-year-old right-hand-dominant boy, who played pitcher on a Little League baseball team since the age of 12, presented for evaluation of an injured thumb. One month earlier and after a baseball game he was not able to actively extend the metacarpophalangeal (MCP) joint of his right thumb. He was otherwise healthy and had no known history of trauma. Upon physical examination, his hands appeared normal except that he was unable to actively extend the right MCP joint (Figure 1). Radiographs were normal (Figure 2). Although there were no tenderness, swelling, or redness in the right hand, the extensor pollicis brevis (EPB) tendon could not be palpated (Figure 3), and the diagnosis of spontaneous rupture of the EPB tendon was made. Surgery was carried out 4 weeks after his first visit because of his chief complaint of difficulty in catching the ball, which interfered with his playing baseball. At operation, a hypoplastic EPB tendon was found ruptured at the level of the carpometacarpal joint of the right hand (Figure 4). The proximal part of the tendon was hardly detectable, and the distal part was very thin with an insertion attached to the extensor hood like the tip of a mop with very fine fibers. During surgery, the MCP joint could not be extended by directly pulling on the end of the distal EPB tendon. Consequently, an extensor indicis proprius (EIP) tendon transfer was carried out (Figure 4). After plaster fixation of his right thumb for 4 weeks, the patient was started on a rehabilitation program. Six weeks after the operation, the patient had full-range movement of his right thumb (Figure 5) and index finger. At 12 weeks he started to grasp the ball, and at 24 weeks he returned to baseball full time.

## Discussion

Rupture of the extensor pollicis brevis (EPB) tendon is rare. Although some isolated extensor pollicis longus (EPL) ruptures are induced by radius fractures and chronic tendonitis (1,2), EPB ruptures have been described only in association with soft-tissue injury of the thumb metacarpophalangeal (MCP) joint (3,4) and a sequential traumatic rupture (5). Batra et al. have reported an unusual case of isolated sequential bilateral EPB rupture in a young man, involving different mechanisms of injury (5). To the best of our knowledge there have been no published reports on spontaneous rupture of the EPB tendon in the English literature. Here we describe a rare case of spontaneous rupture of the EPB tendon induced by the stress of pitching in Little League baseball games. Boyes et al. have shown that the EPB tendon is phylogenetically a new muscle found only in humans and gorillas as a separate muscle (6); other numerous anomalies of the EPB tendon have also been reported (7–9). An awareness of the variations in the anatomy of the EPB is essential, especially that the EPB may be thin or actually absent. Although generally the EPB tendon terminates at the base of the proximal phalanx immediately after passing the MCP joint, various patterns of its insertion have been described (8). In a cadaver study, the rate of absence of the bony insertion at the base of the first phalanx and insertion at the extensor hood has been almost 70% (9). In our case the insertion of the EPB was like a mop, and, interestingly, the extension of the MCP joint was not possible by traction of the EPB tendon during the surgery. Apparently therefore, the spontaneous rupture of this immature EPB tendon was induced by the repeated stress of ball pitching since a very young age. The EPB is considered to play a minor role in the motor function of the thumb. Britto et al. have described two cases in which the tendons of the abductor pollicis longus and the EPB were lost without significant loss of thumb function (10). Furthermore, although the various abnormalities of the EPB described in cadaver studies (9) implied that the EPB tendon need not be reconstructed, in our case the patient wished to undergo reconstruction for full extension of the MCP joint in order to continue playing baseball. At surgery an extensor indicis proprius (EIP) transfer was carried out because the EPB tendon could not be repaired. Transfer of the EIP tendon is both recommended and commonly used in reconstruction of the EPL tendon (1,2), and reports have shown that the outcome of this procedure is good. Furthermore, EIP tendon transfer has also been used in restoring the function of the EPB in bilateral EPB tendon rupture (5). Therefore, transfer of the EIP tendon was selected as a means of reconstructing the function of the EPB in our case. Six months after the surgery, the patient successfully returned to playing baseball. This method is one of the choices for the treatment of unrepairable EPB tendon ruptures in athletes.
